# Overview of findings from a 2-year study of claimants who had sustained a mild or moderate injury in a road traffic crash: prospective study

**DOI:** 10.1186/s13104-017-2401-7

**Published:** 2017-02-01

**Authors:** Bamini Gopinath, Jagnoor Jagnoor, Nieke Elbers, Ian D. Cameron

**Affiliations:** 1 John Walsh Centre for Rehabilitation Research, Sydney Medical School, Kolling Medical Research Institute, University of Sydney, Sydney, Australia; 2 The George Institute for Global Health, Sydney Medical School, University of Sydney, Sydney, Australia; 3 John Walsh Centre for Rehabilitation Research, Kolling Institute, Royal North Shore Hospital, Corner Reserve Road & First Avenue, St Leonards, NSW 2065 Australia

**Keywords:** Inception study cohort, Minor injuries, Claimants, Health outcomes, Return to work

## Abstract

**Background:**

Studies have shown that in people injured in a road traffic crash, persistent symptoms are common and can lead to significant ongoing personal impact. Hence, elucidating factors associated with the human costs are key to reducing the socio-economic burden of road traffic injuries. Therefore, in this study we aimed to track the experience and key outcomes of persons who had sustained mild/moderate injuries as they returned to health (and work, where relevant) following a road traffic crash.

**Results:**

It is an inception study cohort of adults who had sustained mild to moderate injuries (that is, except serious injuries) in motor vehicle crashes in New South Wales, Australia, who were recruited and interviewed at baseline (within 3 months of the crash) and at 6, 12 and 24 months post-injury. We found that minor injuries had major impacts on pain ratings, physical and mental well-being, health-related quality of life and return to work and pre-injury participation during the 24 months post-injury phase. Further, for mild to moderately severe injuries, biopsychosocial factors appear to be prognostic indicators of recovery (not the location or type of injury). Examples of key biopsychosocial factors are: age; preinjury health; quality of life; reactions to injury (catastrophising, and pain); social support and the third party insurance compensation system.

**Discussion:**

This study highlights the considerable impact of apparently “minor” road traffic crash injuries at a population level and suggests targeted approaches to the tertiary prevention of long-term morbidity and disability. Study findings have also reiterated the importance of looking beyond the injury to the ‘whole person’.

**Electronic supplementary material:**

The online version of this article (doi:10.1186/s13104-017-2401-7) contains supplementary material, which is available to authorized users.

## Background

In this report we provide an overview of the findings from a prospective study of persons with mild to moderate injuries sustained in a road traffic crash who had also made claims to the New South Wales (NSW) Compulsory Third Party (CTP) insurance. There is an evidence-base to show that health and social outcomes subsequent to road traffic crash injuries are linked to an individual’s cognitive and psychosocial responses, as well as relationships between the injured person and the compensation systems with which they engage [1, 2]. There is increasing body of research suggesting that the hampered recovery in the compensation process is caused by the compensation process itself [2, 3]. Numerous compensation factors have negative impacts on health, such as claim duration, medical assessments, and lawyer engagement [4, 5]. Interestingly, an under-investigated area is the impact of the interaction with the insurance company, which could be considered to have the biggest effect on claimants’ long-term outcomes. Moreover, most of the epidemiological evidence in this area has come from studies that have been conducted in other Australian states or overseas, and as such; there has been a lack of NSW data in this research area. Further, there is paucity of cohort studies with a long follow-up that have assessed key outcomes and their predictors among participants who have sustained a minor injury in a vehicle crash.

This study includes adults who had sustained mild to moderate injuries (that is, except serious injuries) in motor vehicle crashes in NSW, who were recruited and interviewed at baseline (within 3 months of the crash) and at 6, 12 and 24 months post-injury. The study was funded by the NSW State Insurance Regulatory Authority (SIRA), formerly known as the Motor Accidents Authority. The impetus for SIRA funding this longitudinal study was that they had a limited understanding of the predictors or drivers of the different service and recovery outcomes and pathways. Hence, developing and implementing a prospective study cohort would address this gap in knowledge, as it would help SIRA work towards understanding and improving client outcomes. The questionnaires administered in this study covered a diverse range of content from pre-injury health and employment status to crash-related circumstances, and injury characteristics to post-injury health, psycho-social factors, vocational status and socio-demographic considerations. The key research objectives are summarised as below:

- To establish the health status, vocational status and claimant experience, and describe how they change as they journeyed through the NSW CTP scheme in the first 2 years after the injury;
- Investigate the predictors that may lead to changes in key health and social outcomes among NSW CTP claimants, and how these predictors impact their recovery over the 2 years;
- Establish a uniform set of outcomes data and a uniform data collection method for implementation by insurance schemes;
- To identify important factors that SIRA could focus on during claims management to improve long-term client outcomes; and
- Inform the future design of a larger, prospective study that incorporates claimants as well as non-claimants recruited from multiple sites throughout NSW, and help further develop a collaborative and multi-disciplinary group of researchers.

## Research significance and importance of the problem

Among people injured in a motor vehicle crash, there is evidence to show that persistent symptoms are common even after a ‘minor collision’ [6, 7]. The total cost of road traffic injuries was estimated to be $2.8 billion Australian Dollars (AUD), in NSW [8]. Almost 70% of this cost was associated with disability, costs of medical services, lost productivity and insurance administration. Clearly, establishing the factors associated with human costs is critical to reducing the socio-economic burden of injuries sustained in a road traffic crash [8, 9]. The research to date has suggested that socio-demographic, pre-injury, and health parameters are associated with unfavorable functional and disability outcomes, regardless of the anatomical nature and severity of injury [10].

Several studies have examined recovery after road traffic injuries; although the focus of prior research has typically been on particular injury types such as whiplash associated disorders or orthopaedic injury [11–13]. Further, as insurance and compensation systems vary by jurisdiction, there have been few studies identifying factors related to injury recovery in NSW. There are several limitations in the NSW compulsory insurance scheme, which is fault-based and is principally adversarial, which can lead to delayed payments and high costs [14]. Therefore, if we are to improve insurance scheme outcomes, clarification of factors related to poorer wellbeing following road traffic injuries in a compensable environment is needed to plan and implement more efficacious interventions [15].

## Methods and design

Potential participants were identified from the NSW SIRA Personal Injury Registry database. SIRA is the government regulator of companies providing third party motor vehicle accident insurance in NSW. In NSW, compensation following road traffic crashes is available under a third party insurance scheme, which is compulsory for all motor vehicles owners. People are eligible to lodge a claim if they are injured as a result of a motor vehicle accident. Damages up to $5000 can be claimed regardless of who was at fault in the crash (by lodging an Accident Notification Form). For claims >$5000, lodgment of a personal injury claim form is required, for which the other party’s insurance is liable. Compensation can be paid for economic loss (e.g. loss of wages is paid as lump sum at claim settlement), non-economic loss (e.g. pain and reductions in quality of life if there is significant permanent impairment) and treatment and rehabilitation costs. The injury database consists of people who made claims either through the Accident Notification Form or from the Personal Injury Claim Form. Claimants aged 18 years or older who had sustained road traffic injuries in NSW between March and December 2010 were identified and invited to participate in the study. Participants were excluded if they: (a) sustained severe injuries (severe traumatic brain injury or spinal cord injury; (b) had an injury requiring hospitalization for >7 days; (c) had a New Injury Severity Score (NISS) >8); (d) were unable to complete questionnaires by telephone in English; or (e) if contact could not be initiated within 60 days of the crash.

**Fig. 1:**
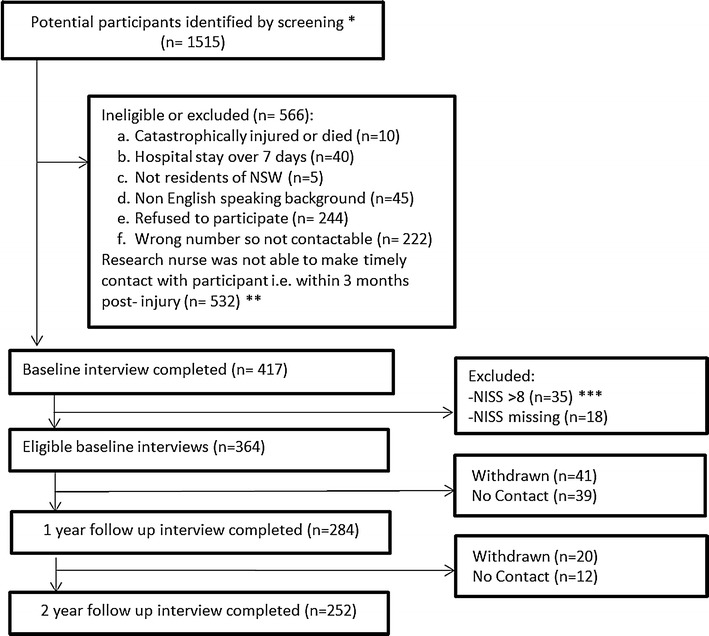
Flowchart of participation in the study.*These participants were identified as potentially suitable for the study as they were screened and excluded if they: (1) had claims associated with death and nervous shock; (2) had severe injuries (defined by the Life Time Care Scheme, NSW i.e. burns, amputation, blindness, spinal cord injury and severe traumatic brain injury); (3) were aged <18; (4) were non-residents of NSW; and (5) were already 3 months post-injury.**Due to the limited time the research nurse was employed on this project, there were participants she was not able to contact for participation within a reasonable time period i.e. 3 months post-injury.***NISS—New Injury Severity Score is determined progressively in the claim process as medical records become available to trained coders at the Motor Accident Authority. Therefore for claims where NISS could not be determined due to insufficient information or score of >8 by 24 months of injury, were excluded from the analysis

A total of 1515 insurance claims that were lodged between March 2010 and December 2010 were identified as potential participants (Fig. 1), and SIRA sent invitation letters to these persons. An opportunity to opt out of the study within 2 weeks was provided, following which, verbal consent was sought. The study was approved by the relevant human research ethics committee. Of the 1515 participants, 854 were ineligible. Of the remaining 661 participants who were eligible, 244 refused to participate. This left 417 who participated at baseline, of these 53 were excluded as they had missing NISS or an NISS >8 (those with severe injury). This left 364 participants included in final analyses. Two-hundred and eighty-four participants (78% of eligible baseline participants) completed 12-month follow-up interviews and 252 (69% follow-up rate) of 364 enrolled and eligible participants completed 24-month follow-up assessments (Fig. 1). Study characteristics of participants versus non-participants (not followed up at 12 or 24 months) were compared. Non-participants versus participants were more likely to be older, male, and have higher mean pain severity scores at baseline.

Study participants were interviewed by telephone on average 56 (range 25–102) days following the crash. The structure interview schedule used a closed format. The interview took around 45 min and all interviews were administered by one trained and experienced research nurse. Information on demographics, return to work, crash details, pain severity, disability and health-related quality of life was collected. The following section provides a summary of questionnaire content by key domains:

### Socio-demographic and personal factors

These factors included: age, gender, height and weight, country of birth, education, occupation, and marital status.

### Return to work status

Vocational status pre-injury was determined based on whether or not the participants was working in a paid job at the time of the crash, nature of the role, and hours worked. These questions were repeated at each follow-up survey post-injury.

### Pre-injury health

Pre-injury chronic pain and any comorbidity prior to the injury were self-reported. General health status prior to the motor vehicle accident was also self-reported, using a five point Likert scale (excellent, very good, good, fair, or poor).

### Injury

- The abbreviated injury scale coding system (based on the NISS) classified participants into: mild (1–3) and moderate (4–8) musculoskeletal injury categories [16]. Trained and experienced coders were used to code the reported injuries. NISS data is obtained progressively in the claim process as medical records become available to trained coders at SIRA. Participants also reported whether they had sustained a whiplash and/or fracture in the accident.
- Hospital admission status following the injury.

### Accident-circumstances

Data on the accident role i.e. whether they were the driver, passenger or pedestrian, and whether they were at fault or not at fault in the accident was obtained from the SIRA Personal Injury Registry database and is based on information determined through the claims process.

### Psychosocial factors

- Disability from musculoskeletal pain impacting on return to work was determined using the 10-item Orebro Musculoskeletal Pain Screening Questionnaire (OMPSQ) [17].
- Pain catastrophising beliefs determined through the use of the pain-related self-statements scale-catastrophizing subscale [18].

### Health

- Global health: using the widely used self-rated health item—short form-12 (SF-12) [19].
- Health-related quality of life: using the EQ-5D which has five dimensions: mobility, self-care, usual activities, pain/discomfort and anxiety/depression [20].
- Pain: overall pain severity was assessed using a 0 (no pain) to 10 (worst pain imaginable) numeric rating scale (NRS) experienced in the past 24 h. Body mass index calculated from self-reported height and weight.

### Compensation scheme experience and legal involvement

- Time to claim closure was established using dates recorded in the SIRA personal injury register database, that is, by subtracting the crash date from the claim settlement date.
- Claim data also collected included whether a prior claim was lodged by the participant, which insurance company dealt with the claim, whether a lawyer was involved, claim settlement date, and dissatisfaction with the claims management and/or the insurance company.
- Participants were also asked to explain their satisfaction or dissatisfaction with the claims management process.

## Results

Data analysis focused on profiling study participants and describing health and social outcomes during the 2 years following the injury. Multivariable analysis also explored the different factors that predict or influence recovery in the 24 months after the crash.

### Health outcomes

#### Pain severity

- In this study, an average pain numeric rating scale (NRS) of 4.5 at 24-month follow-up indicates persistent pain or non-recovery after the injury.
- In multivariable analysis, physical health (SF-12 physical component summary, PCS), pain-related work disability and pain catastrophizing were predictors of persistent pain following minor injuries.
- Injury severity score was not found to be an independent predictor of persistent pain.

#### Health-related quality of life

- Mean SF-12 PCS and mental component summary scores in this study were still below the Australian population norm at 24 months [21].
- In multivariable analysis, socio-demographic indicators (age, marital status and paid employment), pre-injury health factors (chronic illnesses and general health), injury characteristics (presence of whiplash), and bio-psychosocial correlates (e.g. pain severity ratings, pain catastrophising, OMPSQ scores, anxiety/depression) were independent predictors of health-related quality of life, 24 months post-injury.
- Injury severity at baseline was not a prognostic indicator of health-related quality of life at follow-up, after multivariable-adjustment.
- Stratified analysis showed that older versus younger participants who sustained an injury had lower SF-12 PCS and EQ visual analogue scale scores at 24 months, after adjusting for potential confounders [22].
- Sub-group analysis also showed that hospitalization status does not influence long-term health status, after multivariable-adjustment [23].

### Social outcomes

We determined the socio-demographic, psychological, health- and injury-related characteristics that are independently associated with the following social outcomes: (1) return to work, sustained return to work and resuming full-duties at work 12 and 24 months after the injury; and (2) Return to pre-injury participation in daily life activities at 12- and 24-month follow-up. Key findings are summarised below.

#### Return to work

- 82% of claimants with mild to moderate injury (who were working prior to injury) returned to work (RTW) at 24 months.
- Most (89%) had resumed full work duties 2 years later.
- Although, just less than 1 in 2 claimants did not report sustained RTW i.e. were not consistently working throughout the 24 months.
- A range of psychosocial factors were the strongest predictors of RTW in multivariable regression models:
  - Quality of life scales (SF-12, EQ-5D) and OMPSQ scores.
  - Socio-economic factors—age, education.
  - Hospital admission and pre-injury chronic diseases.

#### Return to pre-injury activity and participation

- 85% of claimants with minor injury had returned to pre-injury activity and life participation.
- In multivariable analysis, independent predictors of returning to pre-injury activities at 2-year follow-up included: age, SF-12 PCS and EQ VAS scores.

### Compensation scheme experience and legal involvement

#### Delayed claim settlement and associated factors

- Around one in three claimants still had not settled at the 2-year follow-up.
- Predictors of delayed claim closure:
  - Overweight/obese weight status.
  - Presence of whiplash at baseline.
  - Musculoskeletal pain leading to the development of poor return to work outcomes (higher OMPSQ scores).
  - Consulting a lawyer during the study period.

#### Compensation system related issues

- Dissatisfaction with insurance company associated with anxiety/depressive mood [1].
- Five themes found to be associated with reporting stressful interaction with insurance company [1]:
  - Lack of communication.
  - Delayed or interrupted claims settlement.
  - Problematic treatment approvals.
  - Proving liability/causality.
  - Too much paperwork.

## Strengths and limitations of the study

This study has several noteworthy strengths. First, it is a longitudinal design and thus, we were able to determine the long term changes in health and social outcomes, and baseline predictors. Second, we had a reasonable number of study participants. Third, we collected a vast range of variables such as demographic markers, psychosocial and health measures, and injury- and compensation-related parameters. Nevertheless, our findings need to be interpreted with caution due to study caveats. First, we cannot disregard residual confounding, for example we did not collect data on system generated stressors (e.g. frequency and type of dispute) and personality factors (e.g. self-efficacy, resilience), which could have biased observed associations. Second, our findings are unlikely to be generalizable to all populations, as personal injury schemes are dissimilar; hence, the applicability of our findings to specific jurisdictions (e.g. no-fault scheme) and to the broader injury population is unclear. Third, no objective measures were obtained in our study and all were collected via self-report, and this could have led to inaccuracies as a result of e.g. recall bias. Further, baseline data were collected on average 56 days after the accident, which could have caused some participants to over- or under-estimate their pre-injury health. Therefore, we cannot disregard the possibility that some of the health measures might have improved somewhat by the time baseline data was collected, that is, some recovery might have already occurred by the time participants underwent the baseline survey. Further, around one in four participants examined at baseline were not followed up. This is a relatively sizable proportion of participants who were excluded; hence, we cannot discount the possibility that this missing data could have biased observed associations. Also, we did not have a control group of non-injured participants with which to compare key outcomes with. While the majority of tools administered in the current study were validated, there are a few such as those used to determine pain severity and pre-injury general health which have not been validated, hence, findings related to these measures need to be interpreted with caution. Finally, given that this is an observational study we are not able to establish cause and effect, nor are we able to determine the mechanisms that underlie observed associations (Additional file 1).

### Recommendations and potential next steps

Our findings emphasize and underscore the observation that even minor injuries sustained in a vehicle crash can have long-term impacts on health and social outcomes; and while the substantial impact of severe injuries is already well documented, the impacts of mild or moderate injuries have not been extensively studied. Hence, our study data will provide strong evidence for policy initiatives focussing on how compensation factors could improve health and social outcomes, and CTP scheme efficiency and/or cost effectiveness. Our data also suggest potential targets for studies of tertiary prevention of persistent morbidity and disability following the injury [15]. Some important next steps and possible recommendations stemming from this study are listed below:

#### Health and social outcomes following injury

- Identify people at higher risk of poor outcomes early; and consider targeted early intervention strategies.
- In general, poor physical-health, pain-related work disability and pain catastrophising are negative factors.
- A need to record self-reported quality of life of people injured in motor vehicle crashes routinely was apparent from study findings.
- Return to work is problematic for many people with compensable injuries. Data about return to work status should be collected by claims managers.
- Study findings could lead to future research on modifiable factors like health care utilisation for improved outcomes after a crash.

#### Compensation system related issues

- Routine collection of a range of health parameters by insurers at the time of claim lodgment will improve their knowledge of the claimant’s longer-term health and compensation-risk profile [24].
- A wide range of predictors may need to be considered by insurers to facilitate implementation of interventions that minimize the adverse impact of these factors, and thus, leading to reductions in costs and claim duration [25].

## Summary and conclusions

To understand the burden of injury, robust data on minor injury-related disability outcomes is needed [26, 27]; and this study has contributed valuable data towards achieving this objective. In this cohort of people with compensable personal injury following road traffic crashes, minor injuries were observed to have substantial impacts on pain ratings, physical and mental well-being, quality of life and return to work at 24 months post-injury. The study participants had access to payments for treatment and rehabilitation, and loss of wages [15]. The overall findings from this study have reiterated the importance of looking beyond the injury to the ‘whole person’. Specifically, for mild to moderately severe injuries, biopsychosocial factors appear to be prognostic markers of recovery (not the location or type of injury). Examples of key biopsychosocial factors are: age; pre-injury health; quality of life; reactions to injury (particularly catastrophizing, and pain); social support and the compensation system. Study findings also suggest that “physical” and “psychological” factors co-exist in most claimants and both can impact on recovery trajectories. The insight gained from this study will likely contribute towards claimants needs being better met, largely due to a better understanding of the recovery pathway and the factors that influence this. It is anticipated that because of the findings from this study, that organisations such as SIRA and third-party insurance companies will be able to improve their ability to support those who have been injured in transport accidents.

## Additional file

**Additional file 1.** Participant questionnaire administered in the study.

## Abbreviations

CTP: Compulsory Third Party
MCS: mental component summary
NISS: New Injury Severity Score
NSW: New South Wales
OMPSQ: Orebro Musculoskeletal Pain Screening Questionnaire
PCS: physical component summary
RTW: returned to work
VAS: visual analogue scale
