# Supplementary material for: Overview of findings from a 2-year study of claimants who had sustained a mild or moderate injury in a road traffic crash: prospective study
Source: BMC Res Notes. 2017 Feb 1;10:76. doi: 10.1186/s13104-017-2401-7 (PMC5286861; doi:10.1186/s13104-017-2401-7)
Supplement: Supplementary file 1 — Additional file 1. Participant questionnaire administered in the study. [file 13104_2017_2401_MOESM1_ESM.docx]

**Improving health for people with compensable injuries - phase 1**

**Participant Questionnaire – Initial**

1. Participant ID number ……………………….

2. Today’s date ………………………

3. Name ………………………………………

4. Gender  Male  Female

5. Current age ……………………… Date of birth ………

6. Residential post code

7. Marital status  Single  Married/defacto Divorced/widowed/separated

8. Country of birth ………………………………

9. Language spoken at home

10. Education Postgraduate degree

Graduate diploma or graduate certificate

Bachelor degree

Advanced diploma or diploma

Certificate

Secondary education

Primary education

Pre-primary education

11. Occupation at time of accident ……………………………………….

Occupational Group

Managers or administrators

Professionals

Associate Professionals

Tradespersons or related workers

Advanced clerical or service workers

Intermediate clerical, sales or service workers

Intermediate production or transport workers

Elementary clerical, sales or service workers

Labourers or related workers

12. Hours worked per week (before accident)

13. Work status at time of accident

Paid work

Self employed

Non paid work – voluntary

Student

Home duties

Retired

Unemployed (health reasons)

Unemployed (other reasons)

Other (specify)

14. Health status before accident (1):

Before the motor vehicle accident how would you describe your general health?

Excellent, very good, good, fair, poor

15. Health status before accident (2 - EQ5D):

Here are some simple questions about your health and your quality of life in general. Choose the one best answer in each group below, please indicate which statements best describes your health and your quality of life prior to the motor vehicle accident.

1. Mobility

I had no problems in walking about ❑

I had some problems in walking about❑

I was confined to bed ❑

2. Self-care

I had no problems with self-care ❑

I had some problems washing or dressing myself ❑

I was unable to wash or dress myself ❑

3. Usual Activities

I hade no problems with performing my usual activities ❑

*(e.g. work, study, housework, family or leisure activities)*

I had some problems with performing my usual activities ❑

I was unable to perform my usual activities ❑

4. Pain/Discomfort

I had no pain or discomfort ❑

I had moderate pain or discomfort ❑

I had extreme pain or discomfort ❑

5. Anxiety/Depression

I was not anxious or depressed ❑

I was moderately anxious or depressed ❑

I was extremely anxious or depressed ❑

6. To help people say how good or bad a health state was, we have drawn a scale (rather like a thermometer) on which the best state you can imagine is marked 100 and the worst state you can imagine is marked 0.

We would like you to indicate on this scale how good or bad your own health was prior to the motor vehicle accident, in your opinion. Please do this by giving a number between 0 and 100 that indicates how good or bad your health state was then.

Score …………………………

16. In the three months before the accident how many times did you see a doctor or other health worker?

17. Before the accident did you have a chronic illness (lasting more than 3 months) that had been diagnosed by a doctor?

**Now there are some questions about your health now.**

**18. SF12 (includes SF6D)**

| 1. In general, would you say your health is after the motor vehicle accident:  Excellent 1  Very good 2  Good 3  Fair 4  Poor 5 | | | | | | | | | | |
| --- | --- | --- | --- | --- | --- | --- | --- | --- | --- | --- |
|  | | | | | | | | | | |
| The following questions are about activities you might do during a typical day. Does your health now limit you in these activities? If so, how much? | | | | | | | | | | |
|  | | | Yes, Limited a Lot | | | Yes, Limited a Little | | No, Not limited at All | | |
| **2. Moderate activities,** such as moving a table, pushing a vacuum cleaner, bowling, or playing golf | | | 1 | | | 2 | | 3 | | |
| **3.** Climbing **several** flights of stairs | | | 1 | | | 2 | | 3 | | |
| ***3a Vigorous activities (eg running, lifting heavy objects)*** | | | 1 | | | 2 | | 3 | | |
| ***3b Bathing or dressing yourself*** | | | 1 | | | 2 | | 3 | | |
|  | | |  | | |  | |  | | |
| During the *past week*, have you had any of the following problems with your work or other regular daily activities as a result of your physical health? | | | | | | | | | | |
|  | | | Yes | | | | No | | | |
| **4. Accomplished less** than you would like | | | 1 | | | | 2 | | | |
| **5.** Were limited in the **kind** of work or other activities | | | 1 | | | | 2 | | | |
|  | | |  | | | |  | | |  |
| During the *past week*, have you had any of the following problems with your work or other regular daily activities *as a result of any emotional problems* (such as feeling depressed or anxious)? | | | | | | | | | | |
|  | | | Yes | | | | No | | | |
| **6. Accomplished less** than you would like | | | 1 | | | | 2 | | | |
| **7.** Didn't do work or other activities as **carefully** as usual | | | 1 | | | | 2 | | | |
|  | | | | | | | | | | |
|  | All of the Time | Most of the Time | | A Good Bit of the Time | Some of the Time | | A Little of the Time | | None of the Time | |
| **8.** During the past week, how much did pain interfere with your normal work (including both work outside the home and housework)? | 1 | 2 | | 3 | 4 | | 5 | | 6 | |
|  | | | | |  | |  | | |  |
| These questions are about how you feel and how things have been with you *during the past week*. For each question, please give the one answer that comes closest to the way you have been feeling.  How much of the time during the *past week* . . . | | | | | | | | | | |
|  | All of the Time | Most of the Time | | A Good Bit of the Time | Some of the Time | | A Little of the Time | | None of the Time | |
| **9.** Have you felt calm and peaceful? | 1 | 2 | | 3 | 4 | | 5 | | 6 | |
| **10.** Did you have a lot of energy? | 1 | 2 | | 3 | 4 | | 5 | | 6 | |
| **11.** Have you felt downhearted and depressed? | 1 | 2 | | 3 | 4 | | 5 | | 6 | |
| ***11a Have you been nervous?*** | 1 | 2 | | 3 | 4 | | 5 | | 6 | |
|  | | | | | | | | | | |
|  | All of the Time | Most of the Time | | A Good Bit of the Time | Some of the Time | | A Little of the Time | | None of the Time | |
| **12.** During the past week, how much of the time has your physical health or emotional problems interfered with your social activities (like visiting friends, relatives, etc.)? | 1 | 2 | | 3 | 4 | | 5 | | 6 | |

**19. EQ5D**

Here are some simple questions about your health and your quality of life in general. By the one best answer in each group below, please indicate which statements best describes your health and your quality of life today.

1. Mobility

I have no problems in walking about ❑

I have some problems in walking about❑

I am confined to bed ❑

2. Self-care

I have no problems with self-care ❑

I have some problems washing or dressing myself ❑

I am unable to wash or dress myself ❑

3. Usual Activities

I have no problems with performing my usual activities ❑

*(e.g. work, study, housework, family or leisure activities)*

I have some problems with performing my usual activities ❑

I am unable to perform my usual activities ❑

4. Pain/Discomfort

I have no pain or discomfort ❑

I have moderate pain or discomfort ❑

I have extreme pain or discomfort ❑

5. Anxiety/Depression

I am not anxious or depressed ❑

I am moderately anxious or depressed ❑

I am extremely anxious or depressed ❑

6. To help people say how good or bad a health state is, we have drawn a scale (rather like a thermometer) on which the best state you can imagine is marked 100 and the worst state you can imagine is marked 0.

We would like you to indicate on this scale how good or bad your own health is today, in your opinion. Please do this by giving a number between 0 and 100 that indicates how good or bad your health state is today.

Score …………………………

**20. Orebro Musculoskeletal Pain Questionnaire OMPSQ (short form)**

a. Where do you have pain?

Neck

Shoulder

Arm

Upper Back

Lower Back

Leg

Other (state)

b. How many days of work (or home duties) have you missed because of this injury?

0 days [2]

1-2 days [4]

3-7 days [6]

8-14 days [8]

15-30 days [10]

c. Is your work heavy or monotonous?

0 1 2 3 4 5 6 7 8 9 10

Not at all Extremely

d. How would you rate the pain that you have had during the past week?

0 1 2 3 4 5 6 7 8 9 10

No pain Pain as bad as it could be

e. How much have you been bothered by feeling depressed in the past week?

0 1 2 3 4 5 6 7 8 9 10

Not at all Extremely

f. In your view, how large is the risk that your current pain may become persistent?

0 1 2 3 4 5 6 7 8 9 10

No risk Very large risk

g. Physical activity makes my pain worse.

0 1 2 3 4 5 6 7 8 9 10

Completely disagree Completely agree

h. An increase in pain is an indication that I should stop what I’m doing until the pain decreases.

0 1 2 3 4 5 6 7 8 9 10

Completely disagree Completely agree

i. I should not do my normal work with my present pain.

0 1 2 3 4 5 6 7 8 9 10

Completely disagree Completely agree

j. In your estimation, what are the chances you will be working your normal duties (at work or home) in 3 months.

0 1 2 3 4 5 6 7 8 9 10

No chance Very large chance

**21. WHODAS II**

S. Since the accident how much difficulty did you have in:

None Mild Moderate Severe Extreme / Cannot do

1 2 3 4 5

a. Standing for long periods such as 30 minutes?

b. Taking care of your household responsibilities?

c. Learning a new task, for example. Learning how to get to a new place?

d. How much of a problem did you have joining in community activities (for example festivities, religious or other activities) in the same way as anyone can?

e. How much have you been emotionally affected by your health problems?

f. Concentrating on doing something for ten minutes?

g. Walking a long distance such as a kilometer?

h. Washing your whole body?

i. Getting dressed?

j. Dealing with people you do not know?

k. Maintaining a friendship?

l. Your day to day work?

H2. Overall how much did these difficulties interfere with your life?

H3. Overall in the X past days since the accident, how many days were these difficulties present?

H4. In the X past days since the accident, for how many days were you totally unable to cary out your usual activities or work because of any health condition?

H5. In the X past days since the accident, not counting the days that you were totally unable, for how many days did you cut back or reduce your usual activities or work because of any health condition?

**22. Catastrophising**

Almost never Almost always

0 1 2 3 4 5

a. I cannot stand this pain any longer

b. No matter what I do my pain doesn’t change

c. I need to take some pain medication

d. This will never end

e. I am a hopeless case

f. When will it get worse again?

g. This pain is killing me

h. I can’t go on any more

i. This pain is driving me crazy

**23. Return to Work**

**Was the accident affected your usual work status?**

No

Yes, I am now unable to work at all

Yes, I have returned to work in a reduced capacity

Yes, I have had to change jobs

**AND if you are working, what was the date you returned to work after the accident?**

**Date** ___/___/___

**24. Have you returned to your usual activities?**

Yes

No

**25. Were you admitted to hospital for more than 24 hours after the crash?**

Yes

No

If yes, how many days and which hospital?

………………………………………………………………………………….

**26. How great do you think the danger was that you would die in the motor vehicle accident?**

1. None  2. Small  3. Moderate  4. Great  5. Overwhelming

**The last 6 questions are general questions that may be relevant to a person’s health**

**27. With reference to smoking, what best describes you?**

Current smoker – daily

Current smoker – other (less than daily)

Ex smoker – daily

Ex smoker – less than daily

Never smoked

**28. In the past year, how often did you have a drink containing alcohol?**

Never

Monthly or less

2-4 times a month

2-3 times a week

4 or more times a week

**29. What is your weight in kilograms?**

**30. What is your height in metres or feet?**

**31. Have you made a claim for compensation in the past?**

Yes - once

Yes – more than once

No

**32. Do you have a lawyer for your claim?**  Yes

No

**Thank you for your help with this survey**

**Are there any questions that you would like to ask?**

**We will be in contact in a year**
